# Implantable Port Devices, Complications and outcome in Pediatric Cancer, a Retrospective Study

**Published:** 2016-03-15

**Authors:** H Esfahani, M Ghorbanpor, A Tanasan

**Affiliations:** 1 **Department of Pediatric Hematology/Oncology, Besat Hospital, Hamedan University of Medical Sciences, Hamedan, Iran.**; 2 **Department of surgery, Besat Hospital, Hamedan University of Medical Sciences, Hamedan, Iran.**; 3 **Department of Pediatrics, Besat Hospital, Hamedan University of Medical Sciences, Hamedan, Iran.**

**Keywords:** Adverse effects, Complications, Malignancy, Pediatric, Vascular access device

## Abstract

**Background:**

Peripheral blood vessels, due to availability are used for many years in cancer patients, however in patients with potentially harmful drugs to skin (vesicant drugs) or difficult accessibility to vessels, the use of implantable port (totally implantable venous access port-TIVAP) devices with central vascular access are important.

**Materials and Methods:**

In this retrospective study, 85 pediatric cancer patients younger than 16 years, with TIVAP implantation, were followed for their complications and outcome. In addition to demographic data, patients’ port complications were assessed and compared with published articles.

**Results:**

Mean days of implanted port usage were 531 ± 358 days in all patients. This period was 287 ±194 days in complicated patients. Complications included as infection (tunnel infection and catheter related blood-stream infection), malfunction and thrombosis, skin erosion, tube avulsion, and tube adhesion to the adjacent vessels were seen in 30.6% of patients.

**Conclusion:**

According to the published data and this experience, the most common complications in TIVAP are infection and catheter malfunction. It is important to notice that in order to prolong its efficacious life, effective sterilization methods, prevention of clot formation and trauma, are the most useful measures.

## Introduction

Cancer management, especially venous access is a challenging issue in pediatric patients, but with new techniques and using appropriate methods complications and venous access problems can reduce. The use of intravenous cannula (Angiocatheter) is the standard venous access device in any patients but has some limitation especially in younger cancer patients, whose veins are smaller and more fragile, because of prolong use of vesicant chemotherapeutic drugs and antibiotics. This method predisposes patients to bacterial infections in addition of pain, patients' un-satisfaction and movement restriction of the limbs. One of the most tolerable venous access methods is totally implantable venous access port devices (TIVAP), which are used for more than a decade. These devices are made from a titanium based container covered with a silicon coating and is connected to a pipe that is placed in the main vessels. These devices have many complications which according to the literature, may see in as many as 10 percent of adult patient, and occur early (first month post operation) or late (more than one month). The incidence of complications is different according to their underlying illnesses, which, in adult patients, bronchial cancer have the highest and gastric cancer have the lowest rate (1). The beneficial of this device is not limited to the cancer chemotherapy, but any patients who need prolonged venous access also benefit from this method, however its complications should be in the mind. Catheter dysfunction (thrombotic occlusion) was seen most frequently in some articles, followed by infection (2). Implantation method by the surgeon, sterilization techniques during its usage, the quality and type of needles, long-term care, follow up in patients not using their devices, and thrombosis protection policies are the most important principles of device care. Potential risk factors in TIVAP users which may cause device malfunction are included as infections (exit site infection, tunnel or catheter related infection, pocket infection, blood stream infection) (3, 4), drug extravasations and tissue necrosis, including overlying skin damage and port chamber exposure (5), intraluminal thrombosis and emboli (6) and port fracture and displacement (7). Type and doses of chemotherapeutic agents and leukocyte count, especially neutrophil count during needle insertion, were also considered important, to predispose patients for complication (1,3,4).

## Materials and Method

In this descriptive retrospective study, all children and adolescents patients younger than 16 referred to pediatric oncology department for their cancer during the years 2008 to 2013 were enrolled. Catheter were made by Vygon Company, England, and inserted by a pediatric surgeon under general anesthesia from right subclavian vein. Totally implantable venous access port (TIVAP), are devices made from a titanium base chamber, covered with a silicon coating, settled subcutaneous and access to the main vessels via a flexible hose, in these cases, right subclavian vein. In this experience all devices inserted by single pediatric surgeon, in operation room, after all surgical aseptic techniques and under general anesthesia in pediatric patients (or local anesthesia in adult cases). After surgery, an anterior posterior chest X ray was taken, to examine catheter tip. If the tip of the hose is in subclavian vein, it is all right, but in the case of over insertion, i.e. in the right atrium, surgical correction was carried out. In the cases of non-malignant disorders, or inappropriate chest radiography after surgery, patients were excluded from the study. The port devices were used for venous access and serum therapy, injection of chemotherapeutic drugs and antibiotics, and transfusion of blood, platelets, and plasma, if needed, but not for blood sampling, except in the case of emergency. Patients’ demographic information, such as age, sex, underlying malignancy, complication, duration of port usage and their outcome were recorded and analyzed using the SPSS software version 18 (SPSS Inc., Chicago, IL).

As a definition, catheter-related infection was considered as clinical signs of infection (such as erythema, induration, tenderness and pus formation) in the catheter site, bacteremia and fungemia in the blood collected from the TIVAP, or peripheral blood culture positive, in addition to catheter malfunction. Pressure skin erosion over the port chamber may occur after trauma, skin infection, inappropriate device care, and subcutaneous fat loss in cases of malnutrition, emaciation or severe prolonged chemotherapy related nausea and vomiting. Mechanical problems such as separation of the hose, or port chamber fracture result in lack of proper functioning of the port and drug leakage. Drug extravasation, especially in the case of chemotherapeutic agents may lead to tissue necrosis and eventually followed by infection, may develops by personnel error or port mechanical damages such as trauma. Intraluminal thrombosis resulting in partial or complete blockage of the tube, occur after infection or inappropriate anticoagulation. To determine the correct functioning of the device prior to use and stay open hose after its usage, 10 milliliters of normal saline was injected first, and 10 milliliter of heparin/saline mixture (10 unit heparin per milliliter of normal saline)were used to wash out port chamber and hose.

## Results

**Figure 1:**
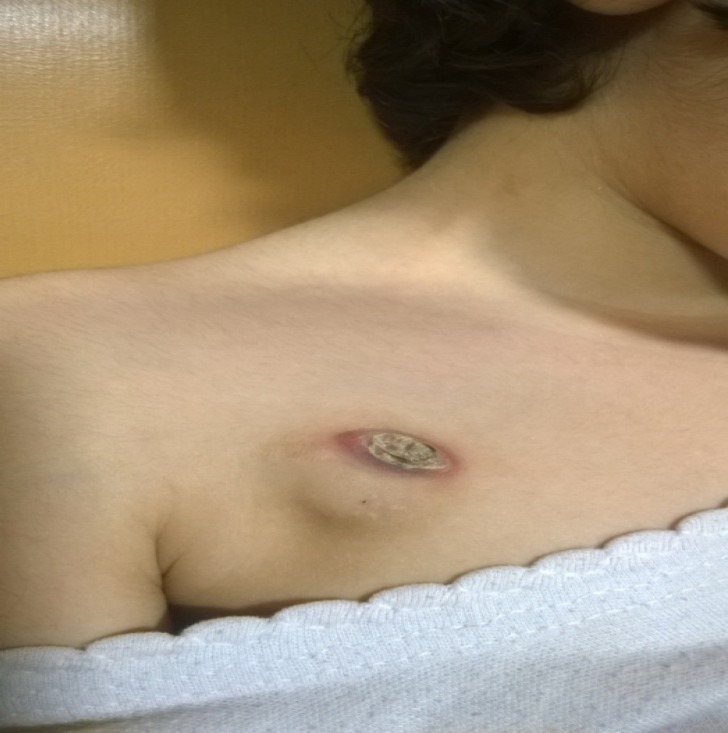
Skin erosion in a four years old girl, treated with surgical correction of the overlying skin

Total numbers of cancer patients diagnosed in the pediatric hematology and oncology ward in Besat hospital, Hamedan, Iran, were 243 patients during the years 2008 to 2013, 85 of them (35%) had been invested in TIVAP. Patients’ age were one to 180 months (mean: 68.09 ± 4.653 months), with female/male ratio of 0.77/1 (female: 43.5%, male: 56.5%). TIVAP complication according to patients’ age, sex and duration of port time are shown in table 1. Percent of complication was calculated as number of complication multiply by 100, divided to total number of totally implantable venous access devices. Devices were fixed in patients as young as three months, to as old as 14 years old. This procedure was done under general anesthesia in the operation room, and by a pediatric surgeon. Mean patients’ age were 69 ± 5.9 months (range: 1-180 months) in uncomplicated cases and 65.9 ± 7.3 months (range: 4-144 months) in complicated cases. The most common baseline malignant condition was leukemia (49.2%), followed by lymphoma (13.6%) and brain tumors (11.9%). Complications were seen most commonly in patients suffered from leukemia (71.5%), followed by germ cell tumor (11.5%) and lymphoma (7.7%). TIVAP related complications were seen in 26 patients (30.6% of total device invested cases). The port remained healthy in patients’ chest for average time of 480 ± 31 days in all patients; this was 287 ± 58 days in complicated and 639 ± 43 days in uncomplicated cases. The most common complication was infection and septicemia (30.8% of complicated cases) followed by malfunction and thrombosis (23.1%), skin erosion (19.2%) (Figure 1), tube avulsion (15.4%) (Figure 1) and vascular adhesion (11.5%) (Figure 3) (Table II).

The ports were removed in 19 patients (22.3%) because of their complications, which can be calculated as 73.1% of device complications. Device removal was also done by the pediatric surgeon, who fixed it, in the operation room and under general anesthesia. Surgical and medical (antibiotic therapy) approaches without its’ removal, were effective in repairing ports in six patients (23% of complicated cases, 11.5% of each surgical and medical approaches). These complications were included as skin erosion included port chamber exposure and localized infection, each in three patients. In one case septicemia lead to patient death (3.8% of complicated cases). This patient was a case of acute lymphoblastic leukemia, during consolidation phase of therapy, developed sepsis after chemotherapy induced severe neutropenia (absolute neutrophil count less than 200). Device complication was calculated in 100 days of device usage as: (number of complications 100 / total number of TIVAP), which were 5.8 per 100 days (table II). TIVAP associated infection was seen in 8 patients (9.4% of all patients). Tunnel infection (positive blood culture collected from the portal tube) was seen in 25% of them, and the other had catheter related blood stream infection (positive blood culture with fever, not responded to parenteral antibiotics), who respond to surgical removal of the port in addition to broad-spectrum antibiotic therapy. There was one patient (12.5%) suffered from fungal infection (aspergillus fumigatus). Other (87.5%) had bacterial infection (4 patients, or 50% of infection cases with gram negative bacillus, included klebsiella and proteous, and 3 or 37.5% with gram positive cocci, included staphylococcus aurous). In a 6 year old patient who developed tube avulsion, direct trauma to the chest was occurred by his sibling. Overlying skin was ecchymotic, and patient suffered from pain and tenderness on the chest wall. He was afebrile and his other vital signs were also normal. Echocardiography did not reveale any significant problem. But at chest radiography, separation of the hose from the port chamber was evident. Surgical removal of the port was done, because of patient non satisfaction, but after one month, a new device system was invested in the opposite side of the chest (left side).

**Tabe I:** TIVAP complication, according to patients’ age, sex, and port time

	Complicated	Un-complicated	Total
Number of patients	26 (30.6%)	59 (69.4%)	85 (100%)
Age (years)	5.52 ± 3.09	5.99 ± 3.69	5.85 ± 11.9
Sex (M/F)	1.2/1 (14/12)	1.2/1 (32/27)	1.2/1 (46/39)
Port time (day)	9.58 ± 9.81 m	21.29 ± 11.07 m	17.71 ± 11.95 m
	287.4 ± 294.3 d	638.7 ± 332.1 d	531.3 ± 358.5 d

**Table II:** Observed complications and their prognosisin TIVAP patients

Complications	Patients NO (%)	Antibiotic therapy(%)	Device Removed (%)	Device Repaired (%)	Expired(%)	Per 1000 device days of use/observation
Infection	8 (100)	3(37.5)	4 (50)	0	1(12)	0.18
Malfunction	6 (100)	0	6 (100)	0	0	0.13
Skin Erosion	5 (100)	0	2 (40)	3 (60)	0	0.11
Tube Avulsion	4 (100)	0	4 (100)	0	0	0.09
Vascular Adhesion	3 (100)	0	3 (100)	0	0	0.07
Total	26 (100)	3	19	3	1	0.58

**Figure 2:**
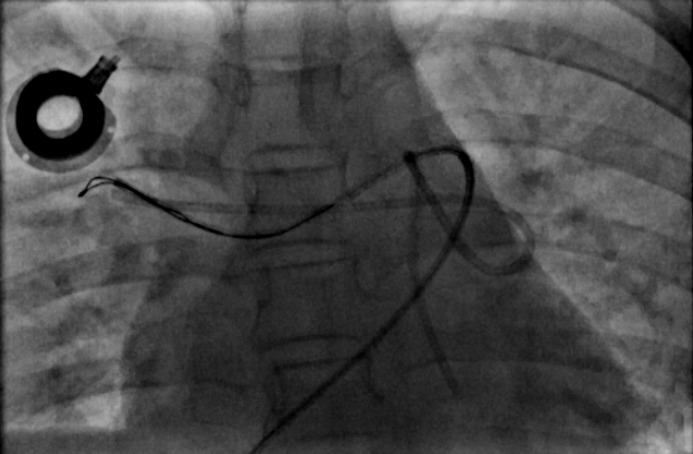
Embolized Port catheter, catched up by snare catheter in the right pulmonary artery, (Anterior posterior view of fluoroscopic angiogram

**Figure 3:**
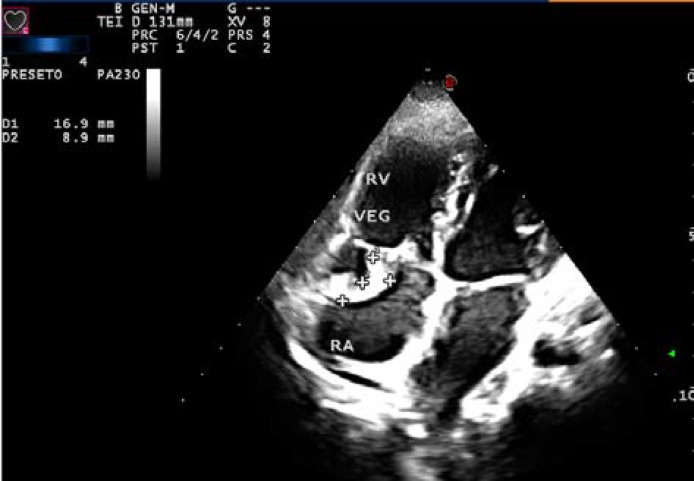
2D Echocardiogram of patient with vegetation in the anterior leaflet of tricuspid valve caused by indwelling port catheter

## Discussion

Rapid and reliable accesses to the blood vessels in patients who require chemotherapy are the main concerns of the oncologist for many years. TIVAP as a reliable method is used by many of the physicians (8, 9). In spite of easy accessibility to the vessels, there are many side effects in these patients that can be attributed to the following, which are mentioned in the literatures: infectious complications of the port, included as exit site infection, tunnel or catheter related infection, pocket infection, blood stream infection and bacteremia without a localized site, overlying-skin necrosis which may lead to overlying subcutaneous tissue loss and subsequently, infection, mechanical port accidents and fracture that may led to move hose port to other tissues (such as pulmonary and carotid vessels) and damage them , intraluminal thrombosis and subsequent port obstruction, tubal avulsion and drug extravasation (4-7, 10).

In the current study, complications occurred in 30.6% of patients that was seen more frequent than literature review. In published articles by Bassi et al and Kock et al, complications were seen in 21% and 13% respectively (3, 11). In the later, catheter-associated infection was seen in 3.2% and thrombosis and portal malfunction were seen in 2.5% of patients, which are significantly lower compare to this study (infection in 9.4% and thrombosis and malfunction in 7% of all patients). Other observed complications were catheter malfunction, migration of the catheter, skin necrosis, catheter fracture, catheter disconnection, and pneumothorax (11). Bassi divided complications to mechanical (non thrombotic, withdrawal malfunction, pinch-off effect and thrombotic occlusion), non mechanical (catheter related blood stream infectious, pocket infection and skin pressure necrosis) and superior vena cava thrombosis. These were seen in 9.87%, 9.87% and 1.2% of patients, respectively. Catheter-associated infection can be directly related with non-compliance with health issues by patients or inappropriate skin decontamination by staff, so it is not out of mind that infectious complications seen more in developing countries. In a study done by Dogar, this complication is directly related to absolute neutrophil count, but this study did not compare these complications. On the other hand, timing of chemotherapy, and its type were also important factor to predispose patients to adverse events, which did not measured in the present study (12). 

Some complications such as overlying skin erosion, which were seen in 5.9% of these patients (5 cases), may occur after its implantation and inappropriate patients care. Other probable causes of this complication are overlying skin infection, drug extravasation and subsequent tissue necrosis, and rapid weight loss (loss of subcutaneous skin fat). Compare to other studies, this was seen more frequent in the present experience that may be due to poor hygiene or lack of adequate care (3, 5, 10). According to this experience, correction of overlying skin erosion, which was successful in 60% of cases, is a reasonable way to maintain the port, if a clear evidence of infectious complications is not in evident.

Malfunction was defined as not fluent fluid passage, which was seen relatively high in this study (7% of TIVAP cases) and led to the removal of the port in all patients. Beforeremoving thedevice, heparinwas usedto eliminatethe possibleblood clot, but unfortunately, was not effective in all cases. In compare to the newer studies, it is more frequent in the old literatures, and was seen in 3.2% to 21.5% of cases, which is more frequent than the present study (3, 11, 13).

Other less frequent but potentially fatal complications was seen in this study tube avulsion and tube adhesion to the adjacent tissues and vessels in 4.7% and 3.5% of cases respectively. These complications did not lead to any permanent disorders due to rapid angiographic approaches and port removal. In spite of low frequency, reports suggests that there occur in cancer patients or usage of improper devices, so routine chest X-ray or device implantation under fluoroscopic guide must be done to determine the status of the device after and during its insertion (7, 14, 15).

Port fracture, drug extravasation and pneumothorax were not seen in the present study, although reported in the literature (5, 7, 10).

## Conclusion

Despite the use of TIVAP as a tool to help in quick and easy access to the central veins of cancer patients for many years, handling of this device requires special attention. Due to the high risk of infection in patients receiving chemotherapy, the points adhere antiseptics in these patients is of particular importance (16). Before commercial use of these devices, staffs’ care patients should be familial with antiseptic agents and methods, in addition to proper anti coagulation, before and after device usage. In addition, patients’ and nurses’ education and proper and right doing the procedures to prevent blood clots in the hose are also very important (17). One of the most important educational programs for patients is to prevent direct or indirect trauma to the chest. Other methods which may use to reduce adverse events, especially during its insertion is fluoroscopic monitoring of the port, an anterior posterior chest radiography just after surgical procedure and before completion of surgery, or cardiac echocardiography to follow the catheter tip.
